# Supplementary material for: Interaction of DDB1 with NBS1 in a DNA Damage Checkpoint Pathway
Source: Int J Mol Sci. 2024 Dec 5;25(23):13097. doi: 10.3390/ijms252313097 (PMC11642328; doi:10.3390/ijms252313097)
Supplement: Supplementary file 1 [file ijms-25-13097-s001.zip › ijms-3278185-supplementary.pdf]

Supplementary Table S1. Top 10 proteins found by mass spectrometry analysis in Nbs1 pull-down.

No	Protein Name	Score	Accession No.	No. of peptides matched
1	Nbs1	710.3	148233328.0	71
2	RAD50 homolog	320.4	238637251.0	32
3	DDB1	110.7	82186503.0	11
4	TUBGCP2	110.3	50415484.0	11
5	Polyubiquitin	70.2	214864.0	7
6	IMB	60.3	4033762.0	6
7	KPNB1	50.2	168693593.0	5
8	GCP3	40.3	21362563.0	4
9	RBBP8	30.1	148227670.0	3
10	LEF1	20.4	148236575.0	2

Supplementary Figure S1

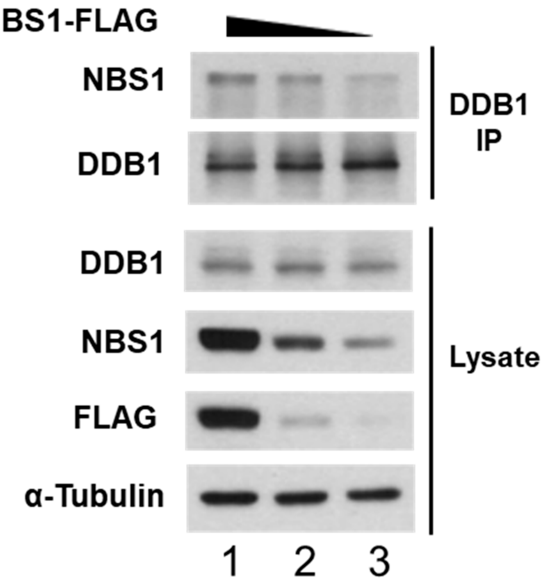

Figure S1. HEK 293T cells were transfected with decreasing concentration of FLAG-tagged human NBS1 (pNBS1-FLAG) vector. Anti-DDB1 IP from cell lysates were performed at 48 h after transfection and immunoblotted with indicated antibodies. The interaction of NBS1 with DDB1 decreased proportionally as expression of NBS1 decreased.

Supplementary Figure S2

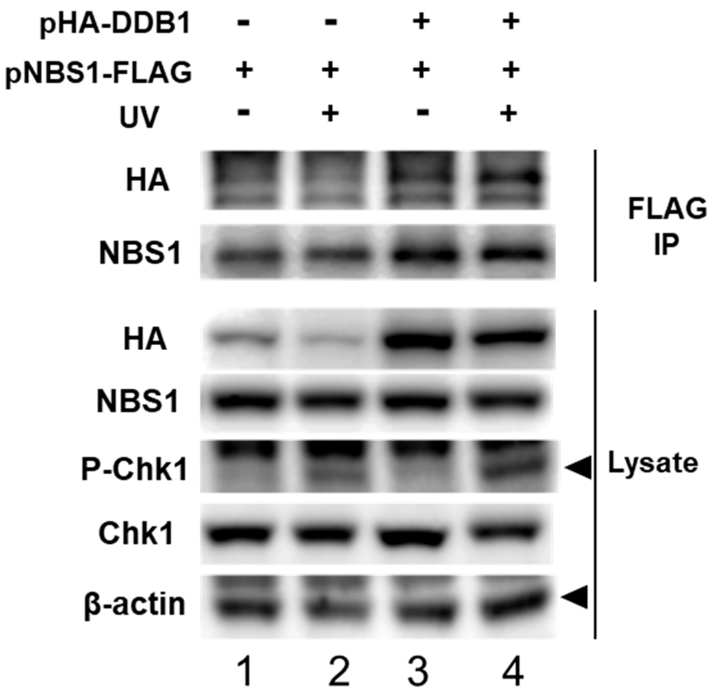

Figure S2. HEK 293T cells were transfected with a control vector or either a vector expressing HA-tagged human DDB1 (pHA-DDB1) and/or FLAG-tagged human NBS1 (pNBS1-FLAG). At 48 h after transfection, cells were mock-treated or exposed to UV (100 J/m^2). Cell lysate were prepared 2 h later. Anti-FLAG immunoprecipitates from cell lysates were immunoblotted with anti-HA and anti-NBS1 antibodies. Cell lysates were immunoblotted with the indicated antibodies. Arrow heads indicate specific bands.

Supplementary Figure S3

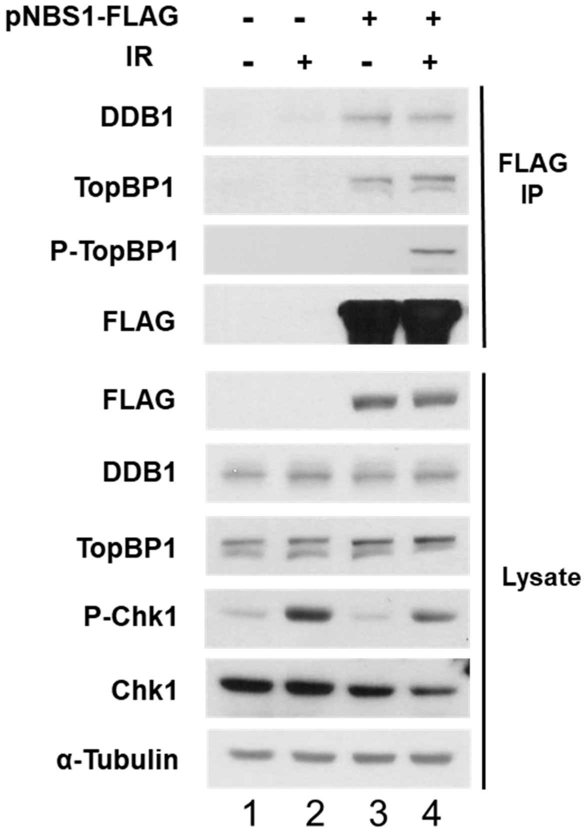

Figure S3. HEK 293T cells were transfected with a control vector or a vector expressing FLAG-tagged human NBS1 (pNBS1-FLAG). At 48 h after transfection, cells were mock-treated or exposed to IR (10 Gy). Cell lysate were prepared 1 h later. After treatment of DNase I with cell lysates, anti-FLAG immunoprecipitations were performed and immunoblotted with anti-DDB1, anti-TopBP1, anti-phospho-TopBP1 and anti-FLAG antibodies. Cell lysates were immunoblotted with the indicated antibodies.

Supplementary Figure S4

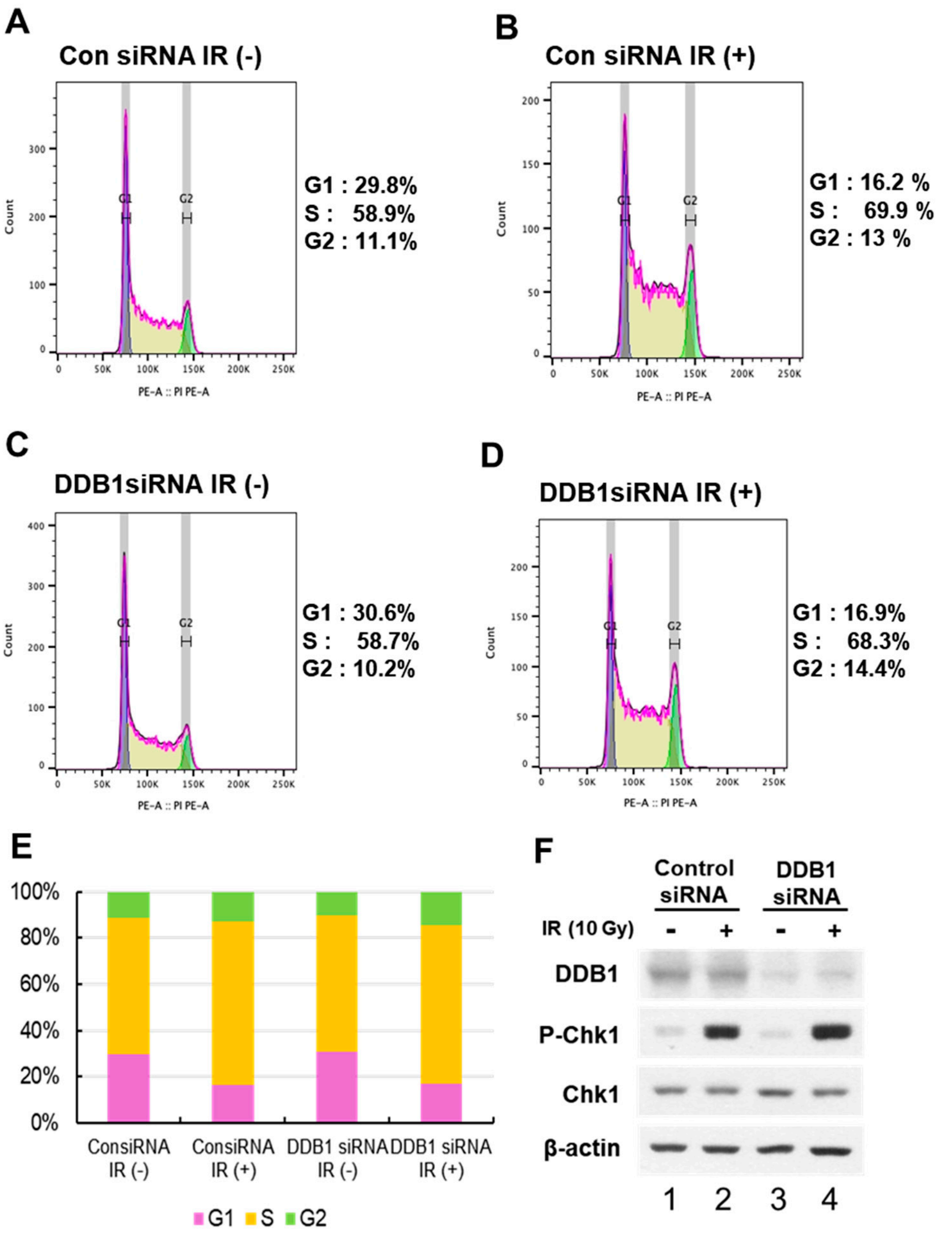

Figure S4. HEK 293T cells were transfected with control or DDB1 siRNA1. At 48 h after transfection, cells were mock-treated (A and C) or exposed to IR (10 Gy) (B and D). Cells were harvested 8 h later and stained with propidium iodide (PI) and subjected to flow cytometry analysis. (F) After either mock-treated or exposed to 10 Gy IR, cell lysates were prepared 2 h later and immunoblotted with the indicated antibodies.

Supplementary Figure S5

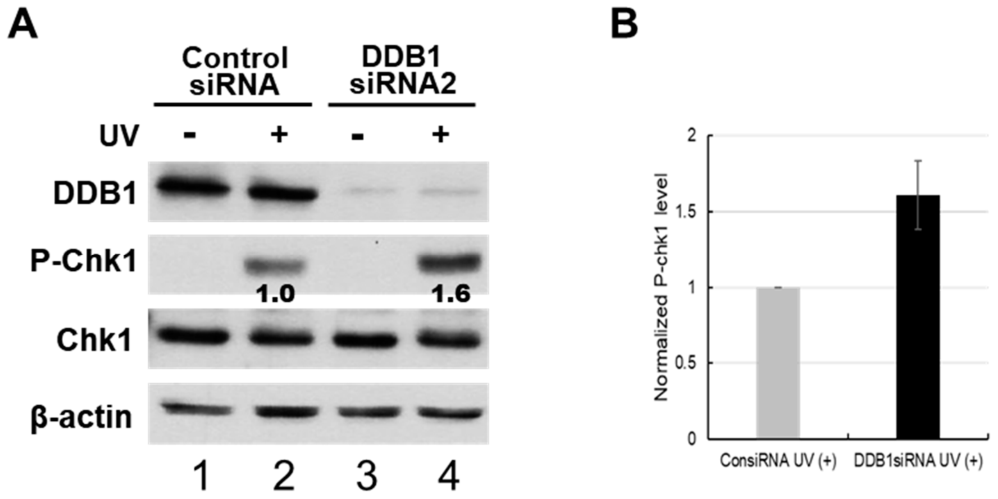

Figure S5. (a) HeLa cells were transfected with control or DDB1 siRNA2. Cells were mock-treated (lanes 1 and 3) or exposed to ultraviolet light (UV) (100 J/m^2) (lanes 2 and 4). Cell lysates were prepared 2 h later and immunoblotted with anti-DDB1, anti-Chk1-P-S317, anti-Chk1, and anti-β-actin antibodies. Quantification of phospho-Chk1 signals (lane 4) normalized to phospho-Chk1 signals (lane 2) are shown underneath individual bands. (b) The graph was obtained from densitometric analysis of immunoblots. The values are normalized P-chk1 levels in DDB1 siRNA treated cells vs control siRNA treated cells. The values are from three independent experiments, and data are expressed as mean ± SD.

Supplementary methods

Immunoprecipitation containing DNase I

HEK293T cells were transfected with indicated plasmid for 48 h and then either untreated or exposed to 10 Gy of IR. Cells were harvested and lysed 1 h after IR with lysis buffer (50 mM Tris-HCl, 150 mM NaCl, 0.5% NP-40, 1% Triton X-100) containing protease inhibitor (PMSF, Pepstatin, Leupeptin, Chymostatin cocktail), $MgCl_2$, DNase I (50 U/mL). Cell lysates incubated 90 min at RT, followed by centrifugation at 13,000 rpm at 4°C and supernatants were collected. Whole cell lysates were incubated with Anti-FLAG M2 affinity gel (Sigma) O/N at 4°C. Bound proteins were immunoblotted with specific antibodies.

Cell cycle analysis

To determine cell cycle distribution, flow cytometry was performed by propidium iodide staining. Cells were trypsinized and washed with PBS and fixed with 70% ethanol. After washing with PBS, cells were incubated with propidium iodide and RNase A and analyzed by BD FACSVerse flow cytometer (BD Bioscience). Data were analyzed using FlowJo software.
